# Transcriptome and genome sequencing elucidates the molecular basis for the high yield and good quality of the hybrid rice variety Chuanyou6203

**DOI:** 10.1038/s41598-020-76762-3

**Published:** 2020-11-17

**Authors:** Juansheng Ren, Fan Zhang, Fangyuan Gao, Lihua Zeng, Xianjun Lu, Xiuqin Zhao, Jianqun Lv, Xiangwen Su, Liping Liu, Mingli Dai, Jianlong Xu, Guangjun Ren

**Affiliations:** 1 Crop Research Institute, Sichuan Academy of Agricultural Sciences, Chengdu, 610066 People’s Republic of China; 2 Institute of Crop Sciences, Chinese Academy of Agricultural Sciences, Beijing, 100081 People’s Republic of China; 3 Sichuan Normal University, Chengdu, 610066 People’s Republic of China

**Keywords:** Genetics, Plant sciences

## Abstract

The yield heterosis of rice is sought by farmers and strong contributes to food safety, but the quality of hybrid rice may be reduced. Therefore, developing new varieties with both high yield and good quality is a heavily researched topic in hybrid rice breeding. However, the molecular mechanism governing yield heterosis and high rice quality has not been elucidated to date. In this study, a comparative transcriptomics and genomic analysis was performed on a hybrid rice variety, Chuanyou6203 (CY6203), and its parents to investigate the molecular mechanism and gene regulation network governing the formation of yield and quality stages. A total of 66,319 SNPs and InDels between CH3203 and C106B were detected in the 5′-UTR, exon, intronic, and 3′-UTR regions according to the reference genome annotation, which involved 7473 genes. A total of 436, 70, 551, 993, and 1216 common DEGs between CY6203 and both of its parents were identified at the same stage in panicles and flag leaves. Of the common DEGs, the numbers of upregulated DEGs between CY6203 and CH3203 were all greater than those of upregulated DEGs between CY6203 and C106B in panicles and flag leaves at the booting, flowering, and middle filling stages. Approximately 40.61% of mRNA editing ratios were between 0.4 and 0.6, and 1.68% of mRNA editing events (editing ratio ≥ 0.8) in CY6203 favored one of its parents at three stages or a particular stage, suggesting that the hypothetical heterosis mechanism of CY6203 might involve dominance or epistasis. Also 15,934 DEGs were classified into 19 distinct modules that were classified into three groups by the weighted gene coexpression network analysis. Through transcriptome analysis of panicles and flag leaves in the yield and quality stages, the DEGs in the green-yellow module primarily contributed to the increase in the source of CY6203 due to an in increase in photosynthetic efficiency and nitrogen utilization efficiency, and a small number of DEGs related to the grain number added spikelet number per panicle amplified its sink. The balanced expression of the major high-quality alleles of C106B and CH3203 in CY6203 contributed to the outstanding quality of CY6203. Our transcriptome and genome analyses offer a new data set that may help to elucidate the molecular mechanism governing the yield heterosis and high quality of a hybrid rice variety.

## Introduction

Rice is one of the world's major crops and is the staple food of more than half of the world's population. Successful application of rice heterosis significantly increased yield. In the past hundred years, the heterosis of crops with distinct yield advantages has been widely used in corn, sorghum, rape, and other crops. Researchers have frequently attempted to reveal the genetic mechanism of heterosis, and have proposed several classical genetic models, such as dominance, over-dominance, epistasis, and non-additive gene expression^1^. However, the molecular mechanism governing heterosis has not been elucidated.

At the DNA and mRNA levels, genomic and transcriptome analysis represent a useful method to study the genetic mechanism of heterosis. Bao et al.^2^ identified 595 upregulated and 25 downregulated tags in LYP9 using the serial analysis of gene expression technique and found that most of the genes were related to enhancing carbon and nitrogen assimilation. Wei et al.^3^ detected 3926 differentially expressed genes (DEGs) between hybrid F_1_, LYP9, and its parents (DEG_HP_) and between the parents (DEG_PP_) and found that the DEGs in the categories of energy metabolism and transport were enriched in DEG_HP_, rather than in DEG_PP_. Huang et al.^4^ believed that numerous superior alleles, such as *Hd3a*, *OsSPL14*, *Gn1a*, *Waxy*, *ALK*, and *qSW5*, exhibiting positive dominance, contributed to heterosis. Chen et al.^5^ concluded that the overdominant effect probably contributed to the grain number heterosis of WFYT025. The genome-wide gene expression profiles were compared to identify the genetic basis of yield heterosis of such species maize^6,7^, *Bombyx mori*^8^, rubber tree^9^, and oil palm^10^. These studies all showed that the DEGs and major genes contributed to heterosis.

The yield heterosis of rice is sought by farmers, but the quality of hybrid rice is reduced. The poor eating and cooking quality of most hybrid rice, especially *indica* hybrid rice, is mainly attributable to a high amylose content (AC), hard gel consistency (GC), and high gelatinization temperature (GT)^11–13^. Recently, we released a new hybrid rice variety, Chuanyou6203 (CY6203), which has the same eating and cooking qualities as the Thai rice variety. The quality of CY6203 conforms to the second grade of the State Standard of the People's Republic of China (Rice)(GB1354-2009). After the variety Chuanyou6203 (CY6203) was released, it was welcomed by farmers. And its planted area had quickly reached 200,000 hectares in rice area of southwest China. In this study, a transcriptome analysis of the panicles and flag leaves of the hybrid rice CY6203 and its parents in the formation of yield and quality stages was performed to identify the molecular modules related to yield heterosis and high quality. The data presented in this report may be help to elucidate the molecular mechanism governing yield heterosis and the formation of high quality.

## Results

### Performance and heterosis of Chuanyou6203

**Figure 1:**
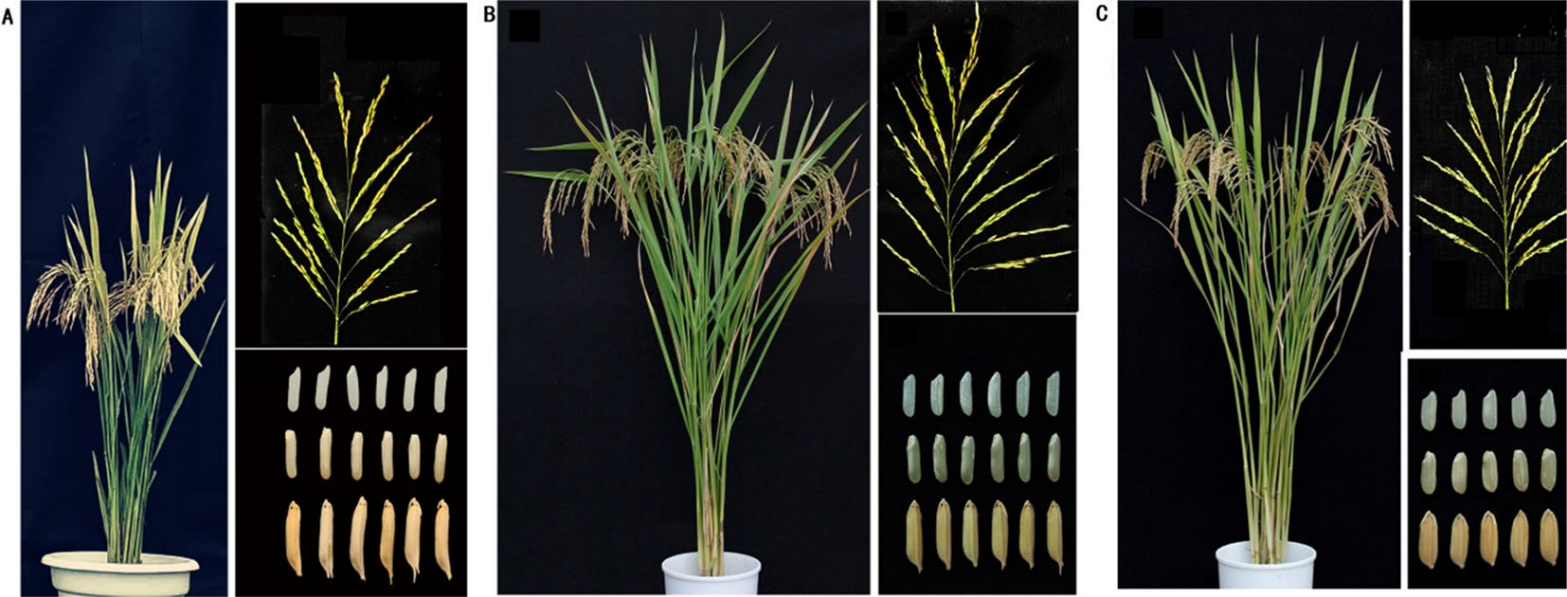
Plant phenotype, panicle and grain shape of hybrid rice variety Chuanyou6203 and its parents. (**A**), (**B**), (**C**) are Chuan106B, Chuanyou6203 and Chenghui3203, respectively.

**Figure 2:**
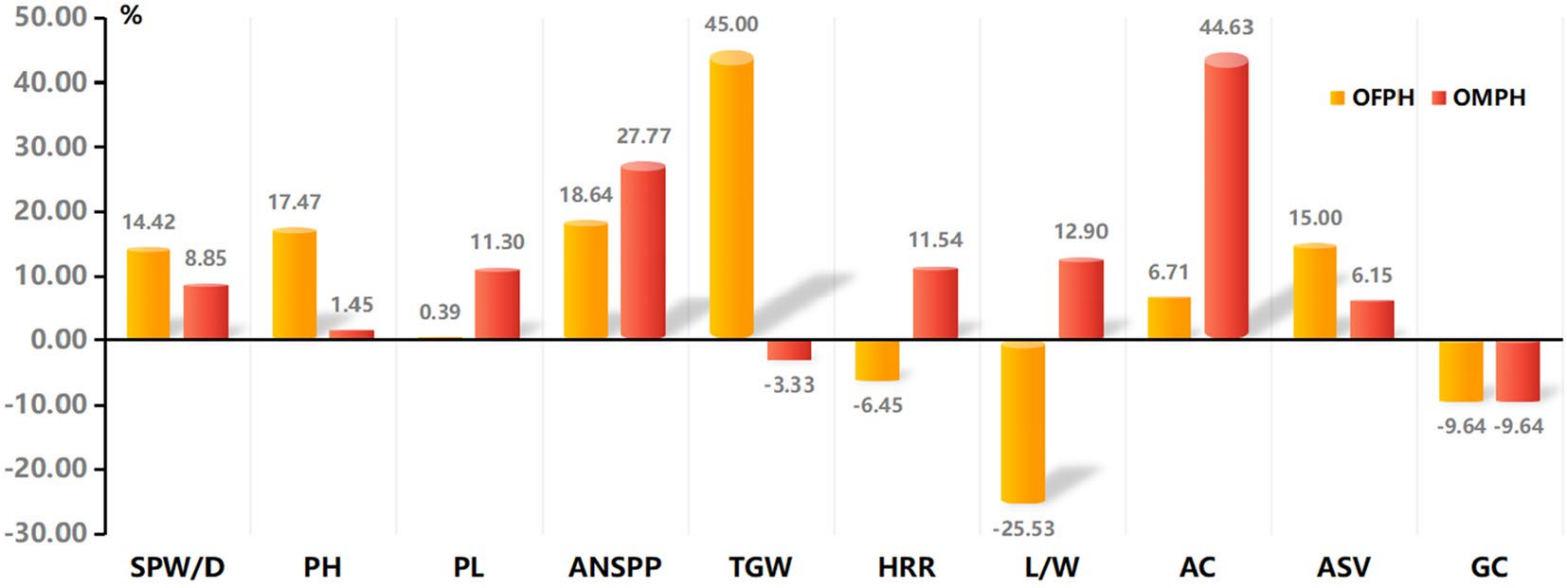
Over-male-parent heterosis (OMPH) and Over-female-parent heterosis (OFPH) of the main agronomic traits and quality traits. SPW/D, single plant weight per day; PH, plant height; PL, panicle length; ANSPP, average number of spikelets per panicle; TGW, 1000-gains weight; HRR, head rice ratio (ratio of head milled rice); L/W, grain length/grain width; AC, amylose content; ASV, alkali spreading value; GC, gel consistency.

Figure 1 shows the plant type, panicle type, and grain shape of hybrid rice variety CY6203 and its parents. We investigated a total of ten traits, including five agronomic traits (SPW/D, PH, PL, ANSPP, and TGW) and five quality traits (HRR, L/W, AC, ASV, and GC) (Table 1). The results showed that there was a high significant difference (*p* < 0.01) between C106B and CH3203, except for ASV and GC, and a significant difference (*p* < 0.05) in ASV. Between CY6203 and its parents, there was a high significant difference (*p* < 0.01) except for PL, TGW, and ASV. There was a high significant difference (*p* < 0.01) between CY6203 and C106B in TGW and ASV, respectively. In PL, a high significant difference (*p* < 0.01) was found between CY6203 and CH3203, and there was no significant difference between CY6203 and C106B. The values of over-female-parent heterosis (OFPH) ranged from − 25.53% (L/W) to 45% (TGW) (Fig. 2). Of these values, OFPHs of agronomic traits were all positive. However, OFPHs were only detected positively in two quality traits, AC (6.71%) and ASV (15%), while strongly negative OFPHs were identified in the other three quality traits HRR (− 6.45%), L/W (− 25.53%), and GC (− 9.64%). The values of over-male-parent heterosis (OMPH) ranged from − 9.64% (GC) to 44.63% (AC). Positive OMPHs were observed in SPW (8.85%), PL (11.3%), ANSPP (27.77%), HRR (11.54%), AC (44.63%), and ASV (6.15%). In contrast, the intensities of OMPHs and OFPHs were remarkably different in PH, PL, TGW, HRR, AC, and L/W, but similar in SPW/D, ANSPP, ASV, and GC. The results suggest that the high yield of CY6203 should be primarily determined by heterosis in SPW/D and ANSPP, while its high quality maybe attributable to the balance among traits AC, ASV, and GC.

**Table 1:** Phenotypic analysis of hybrid rice CY6203 and its parents.

Traits	SPW/D (g/d)	PH (cm)	PL (cm)	ANSPP	TGW (g)	HRR (%)	L (W)	AC (%)	ASV (Grade)	GC (mm)
C106B	0.215 ± 0.002	95 ± 2.15	25.5 ± 0.54**	140 ± 5.31**	20 ± 1.12	62 ± 1.35**	4.7 ± 0.15**	16.4 ± 0.64**	6 ± 0.1	83 ± 2.5
CH3203	0.226 ± 0.002**	110 ± 3.71**	23 ± 1.5	130 ± 2.58	30 ± 2.45**	52 ± 1.37	3.1 ± 0.15	12.1 ± 0.75	6.5 ± 0.1*	83 ± 1.5
CY6203	0.246 ± 0.001**^ab^	111.6 ± 1.9**^ab^	25.6 ± 0.63**^b^	166.1 ± 6.12**^ab^	29 ± 2.5**^a^	58 ± 1.33**^ab^	3.5 ± 0.05**^ab^	17.5 ± 0.48**^ab^	6.9 ± 0.15**^a^	75 ± 1.8**^ab^

SPW/D: single plant weight per day; PH: plant height; PL: panicle length; ANSPP: average number of spikelets per panicle; TGW: 1000-gains weight; HRR: head rice ratio (ratio of head milled rice); L/W: grain length/grain width; AC: amylose content; ASV: alkali spreading value; GC: gel consistency. ^*,**^Significant difference with *p* < 0.05, 0.01, respectively. ^a,b^Significance between CY6203 and C106B or CH3203.

### Genomic variation between two parents of Chuanyou6203

**Figure 3:**
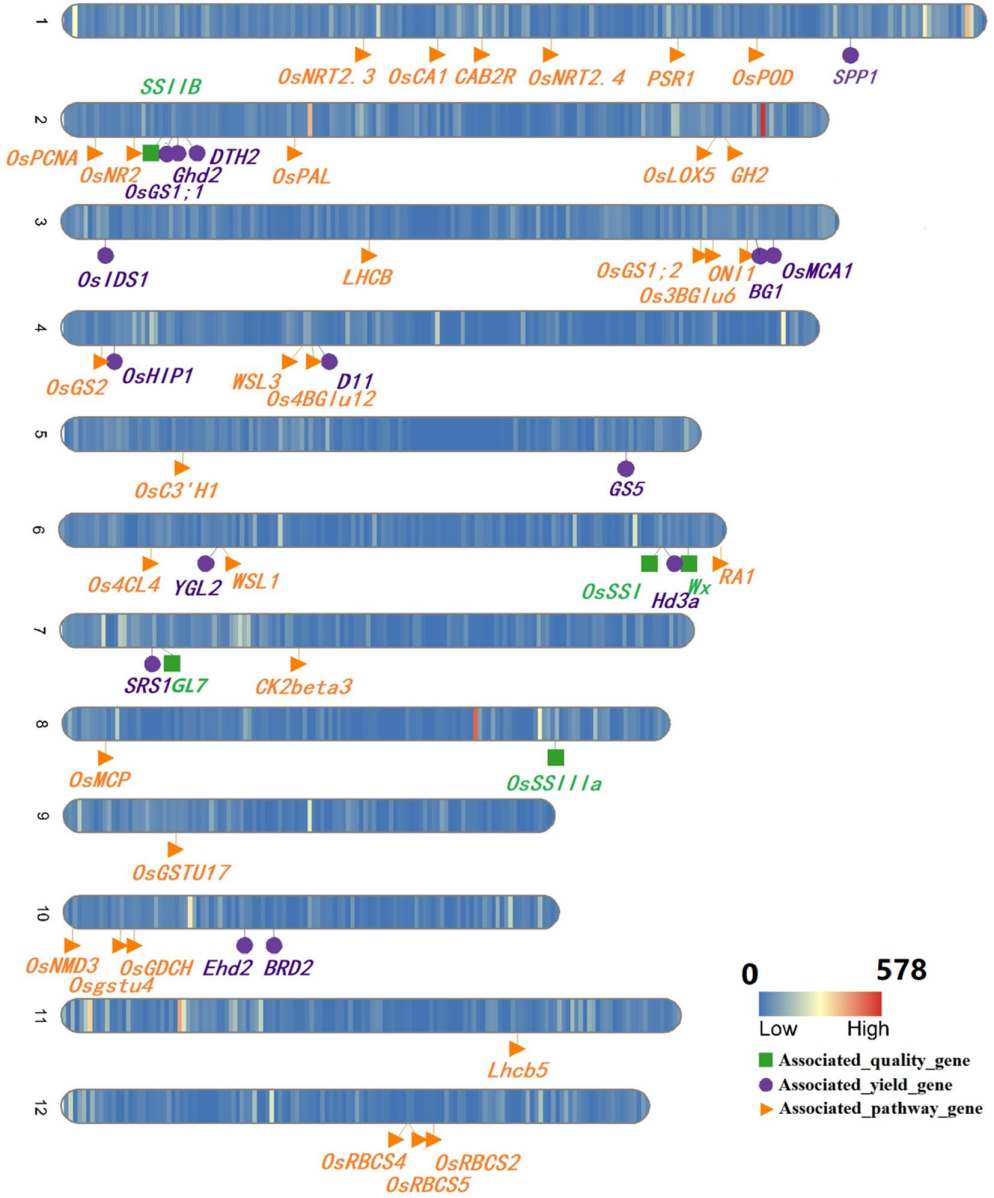
Density of different SNPs and InDels in Chenghui3203 and Chuan106B genomes and their different cloned yield-, quality- and KEGG pathway-associated genes.

Using the Illumina-platform, a total of ~ 175 million and ~ 65 million paired-end clean reads measuring 150 bp in length were generated with average coverage of ~ 61 × and ~ 22 × for the genomes of CH3203 and C106B, respectively (Supplementary Table S1). The GC contents of CH3203 and C106B were 43.29% and 40.73%, respectively. The high-quality reads of each parent were aligned to the Nipponbare reference genome MSU RICE GENOME ANNOTATION PROJECT RELEASE 7 (MSU 7), and their mapped ratios were 94.98% and 92.63%. A total of 66,319 SNPs and InDels between CH3203 (34,102) and C106B (32,217) were detected in the 5′-UTR, exon, intronic, and 3′-UTR regions according to the reference genome annotation (Fig. 3 and Supplementary Table S2), which involved 7473 genes. The average number of variations per gene was 8.87, and the average number of variations per 200 Kb was 35.47. Although most of the variations were randomly distributed in 12 chromosomes, there were several hot-spot regions with high-density variations (mean value = 385.4) on chromosomes 1, 2, 4, 8, 10 and 11. On 3,000,001 bp-3200000 bp of chromosomes 2, the number of variations per 200 Kb was up to 578.

### Gene expression profiling of Chuanyou6203 and its parents

**Figure 4:**
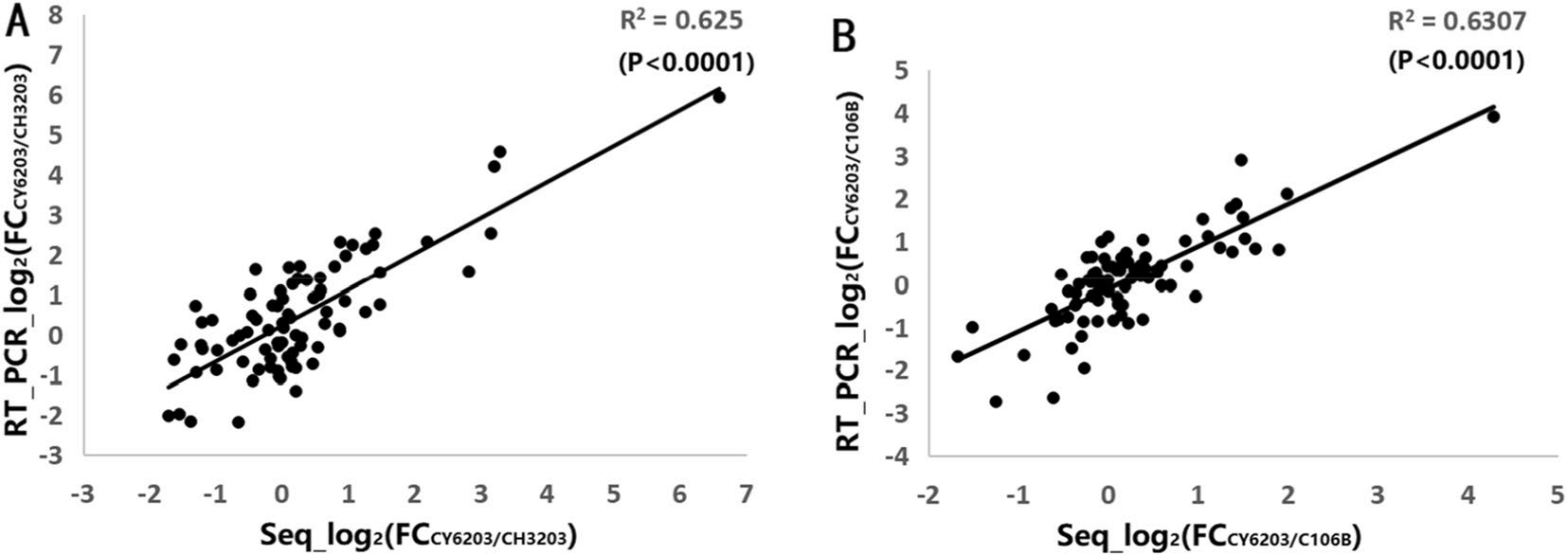
Comparison of the log_2_(FC) of 17 randomly selected transcripts using RNA-Seq and qRT-PCR in Chuanyou6203/Chenghui3203 and Chuanyou6203/Chuan106B.

We performed RNA sequencing and obtained an average of 67,645,949 high-quality clean reads for each of the 45 samples after the removal of rRNA and low-quality reads (Supplementary Table S3).The mean ratio of the high-quality reads mapped to the reference genome was 82.80%, ranging from 77.59 to 86.61%, and an average of 26,344.67 mapped unique genes per sample was identified simultaneously. The significantly positive correlation (*R*^2^ = 0.625, *R*^2^ = 0.631, *P* < 0.0001) between the RNA-seq and qRT-PCR using the value of Log_2_FC_CY6203/CH3203 or C106B_ of 18 randomly selected genes indicated the accuracy and reliability of the RNA-seq results (Fig. 4 and Supplementary Table S7). Then, a total of 39,503 differentially expressed genes (DEGs) were detected among all samples (Supplementary Table S4). Based on the hierarchical clustering of these samples using the FPKM value (Supplementary Figs. S1 and S2), two discrete samples E6203-2 and Y6203-1 were excluded.

**Figure 5:**
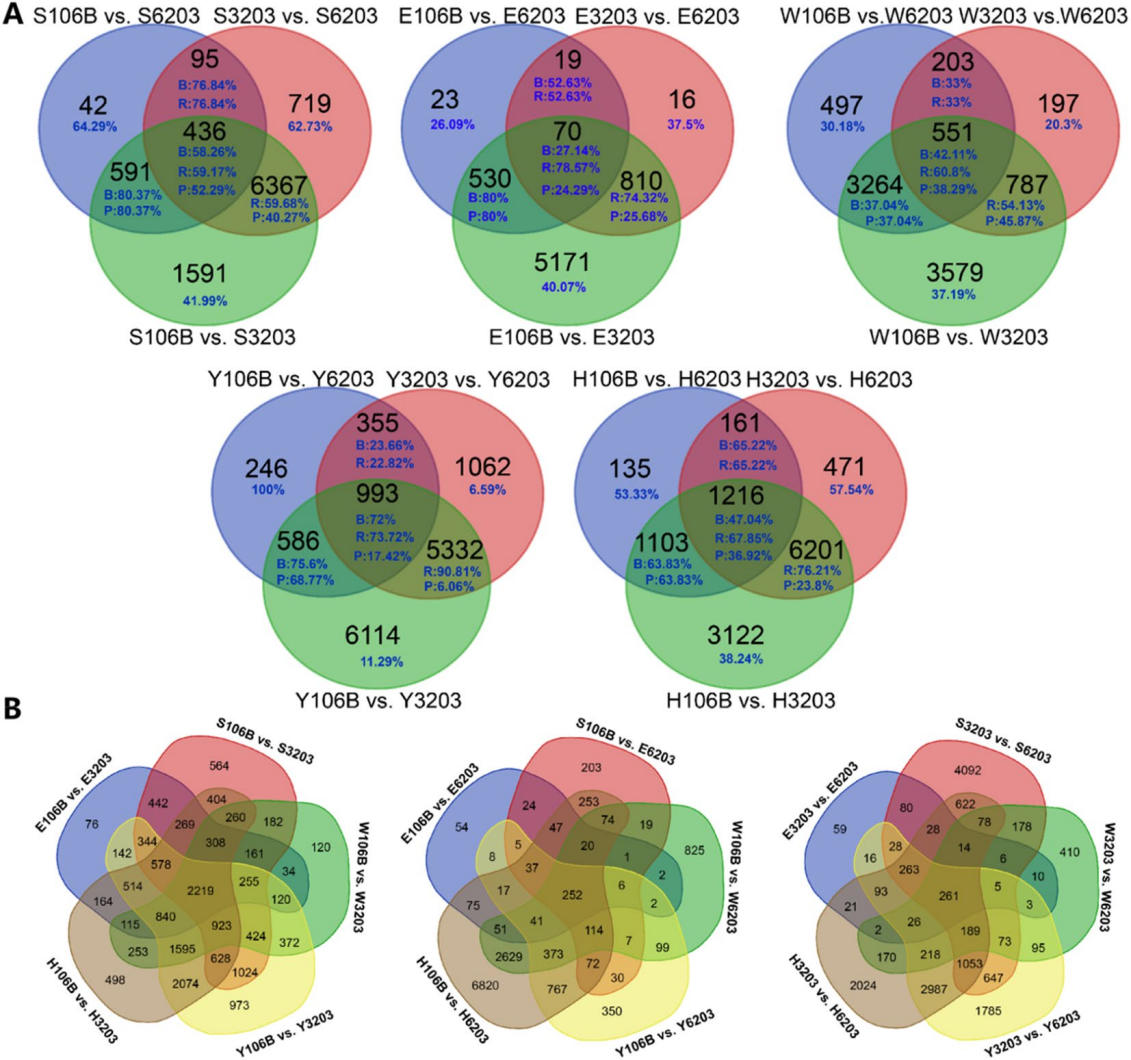
DEGs in hybrid rice CY6203 and its parents. (**A**) Venn diagrams of DEGs between CY6203 and its parents in panicles and flag leaves. (**B**) Venn diagrams of DEGs at each stage. Blue font represents upregulated DEGs, B: C106B vs. CY6203, R: CH3203 vs. CY6203, and P: C106B vs. CH3203; S: Panicle samples7 days before heading. E: Panicle samples 3 days after heading. W: Panicle samples 15 days after heading. Y: Sward leaf samples 7 days before heading. H: Sward leaf samples 3 days after heading.

There were 1164 (42 + 591 + 95 + 436), 642 (23 + 530 + 19 + 70), 4515 (497 + 3264 + 203 + 551), 2180 (246 + 586 + 355 + 993), and 2615 (135 + 1103 + 161 + 1216) DEGs between CY6203 and C106B, and similarly 7617, 915, 1738, 7742 and 8049 between CY6203 and CH3203 in panicles and flag leaves at the booting, flowering, and middle filling stages, respectively (Fig. 5A). Among these DEGs between CY6203 and C106B, there were 829 (27 + 475 + 73 + 228) (829/1164 × 100% = 71.22%), 459 (71.5%), 1,658 (36.72%), 1,488 (68.26%), and 1,453 (55.56%) upregulated DEGs, respectively. Among these DEGs between CY6203 and CH3203, there were similarly 4,582 (60.15%), 673 (73.55%), 868 (49.94%), 5,662 (73.13%), and 5927 (73.64%) upregulated DEGs at the booting, flowering, and middle filling stages, respectively. At the booting stage, the number of DEGs between CY6203 and CH3203 was greater than that of DEGs between CY6203 and CY106B, but the ratio of upregulated common DEGs between CY6203 and C106B was almost the same (58.26% vs. 59.17% and 72% vs. 73.72%) as that of upregulated common DEGs between CY6203 and CH3203. At the middle filling stage, the phenomenon was opposite and the ratio of upregulated common DEGs between CY6203 and CH3203 was higher than (60.8% vs. 42.11% and 67.85% vs. 47.04%) that of upregulated common DEGs between CY6203 and C106B. The number of common DEGs between C106B and CH3203 in panicles and flag leaves at the booting, flowering, and middle filling stages was greater than that of common DEGs between CY6203 and its parents (Fig. 5B). In addition, a total of 436, 70, 551, 993, and 1,216 common DEGs between CY6203 and both of its parents were identified at the same stage in panicles and flag leaves. Of the common DEGs, the numbers of upregulated DEGs between CY6203 and CH3203 were all greater than those of upregulated DEGs between CY6203 and C106B in panicles and flag leaves at the booting, flowering, and middle filling stages.

**Figure 6:**
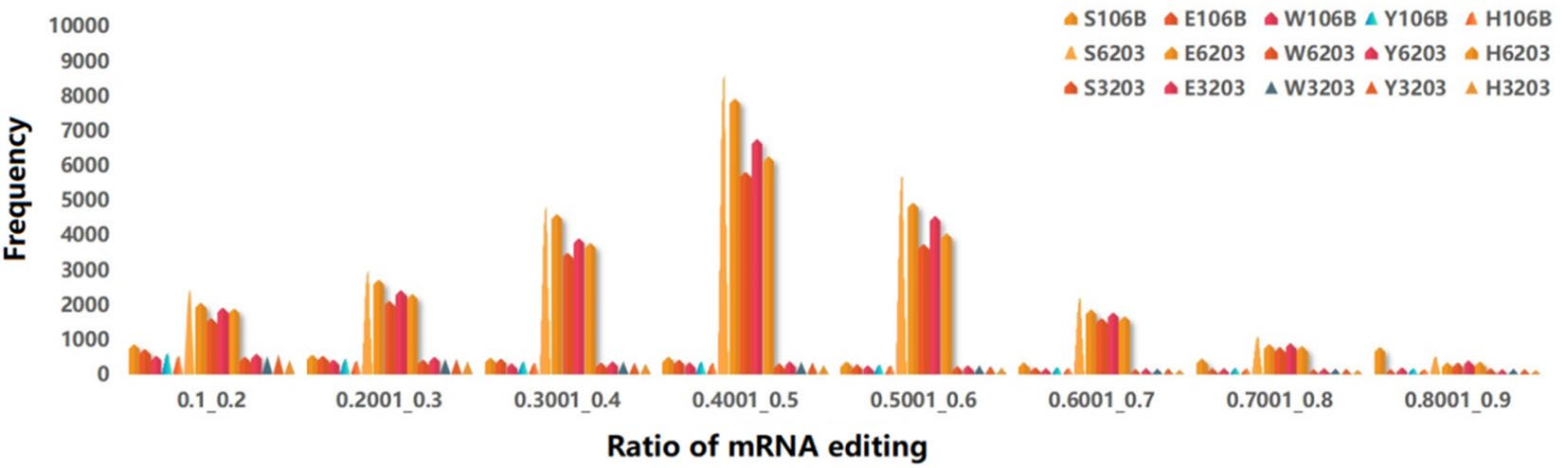
Distribution of frequency of mRNA editing ratios among hybrid rice CY6203 and its parents in leaves or panicles.

The frequency of mRNA editing ratios of each sample of CY6203 followed normal distribution (Fig. 6 and Supplementary Table S5). The average frequency of mRNA editing loci of 15 samples of C106B was 8.13 per Mb, and that of CH3203 was 7.94 per Mb, and that of CY6203 was 63.05 per Mb. The results showed that a great quantity of mRNA editing phenomena occurred in CY6203 due to its heterozygosity, and most mRNA editing ratios were approximately equal to 0.5. Though CH3203 and C106B were pure lines, a small amount of mRNA editing phenomena occurred. For example, the mRNA editing ratio of the *Wx* gene was an average of approximately 0.5119 in an SNP site (T_1768724_ to C_1768724_) of exon 9 in CY6203, while it was an average of approximately 0.8144 in C106B.

### Identification of coexpressed gene network modules

We remove no significant DEGs between F_1_ (CY6203) and its parents (C106B and CH3203) in panicles and flag leaves at booting, flowering, and middle filling stages. The 17,105 DEGs were used to construct coexpression modules by WGCNA^14^. A total of 15,934 DEGs were classified into 19 distinct modules (*R*^2^ = 0.85, *β* = 18) (Supplementary Fig. S3), which were generated by a hierarchical clustering tree. Among these DEGs, discrete DEGs were classified into the grey module. The number of DEGs in the 18 functional modules ranged from 39 (light green module) to 2996 (turquoise module). When the Euclidean distance was 1.0, 19 modules were divided into three groups (Supplementary Fig. S3D). Grey 60 (88 genes), yellow (1574 genes), green-yellow (298 genes), and midnight blue (186 genes) modules were merged into one group, and tan (292 genes), red (1198 genes), black (706 genes), pink (673 genes), blue (2905 genes), and purple (337 genes) modules were combined into another group, and the remaining modules were classified into the other group.

The expression heatmap of each module and expression histogram of corresponding eigengenes showed that the eigengenes were regularly expressed at different levels at the different stages or materials (Supplementary Fig. S4). For example, blue and turquoise modules showed upregulation at the booting and heading stages in panicles. The brown module showed upregulation in CY6203 compared with CH3203, except for the middle filling stage. The green-yellow module showed that eigengenes were higher in CY6203 than C106B. Among the 18 modules, few eigengenes showed higher expression in CY6203 than its parents at the three stages.

### GO and KEGG pathway enrichment in coexpression network modules

**Figure 7:**
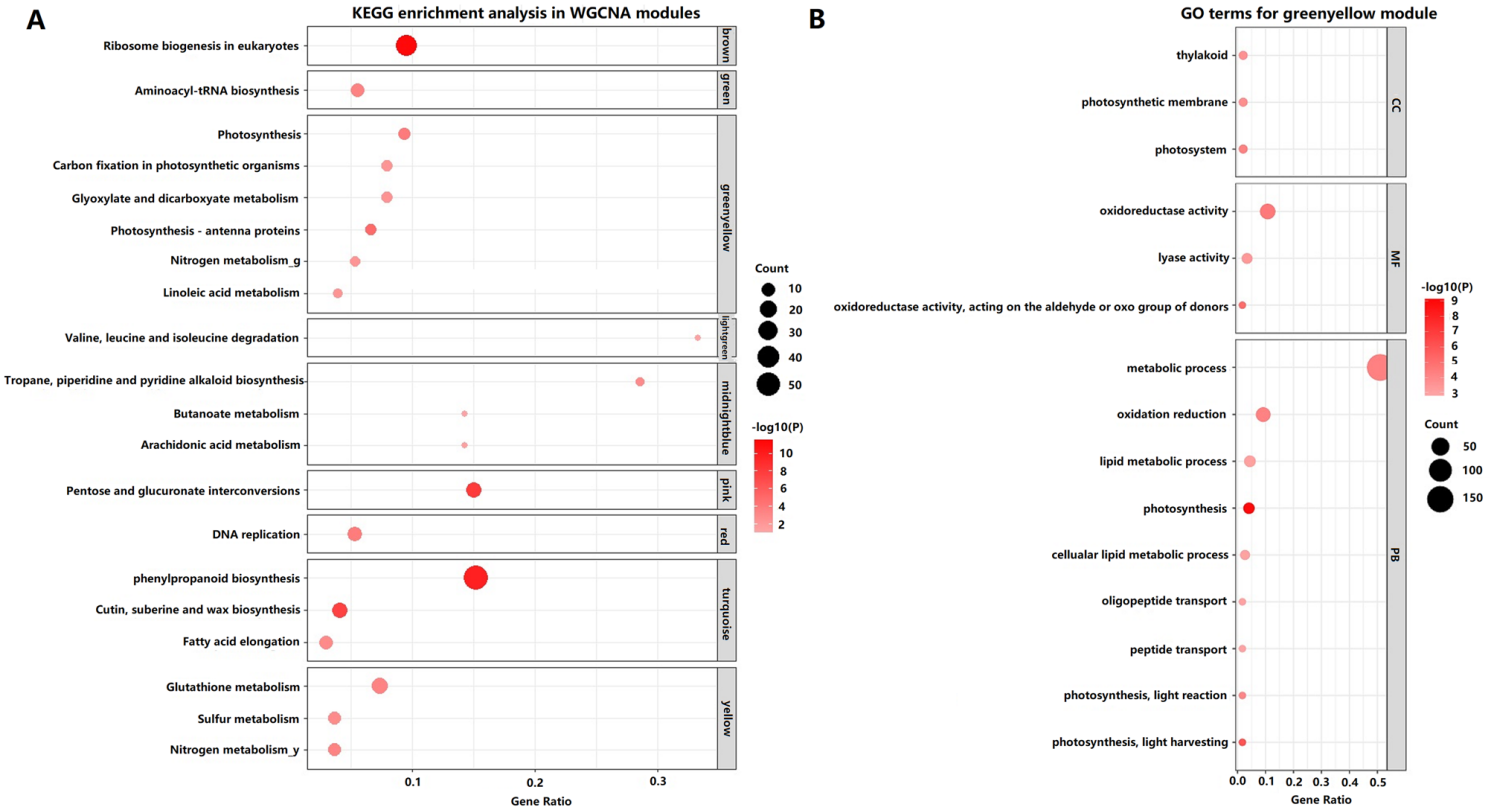
KEGG pathway significantly enriched in WGCNA modules (**A**) and GO terms significantly enriched in green-yellow module (**B**). The size of the bubble indicates the number of genes in each module. The colour of the bubble indicates a significance level for a KEGG pathway or GO term group of genes.

A total of 219 DEGs of 9 modules were significantly enriched in 20 KEGG pathways (Fig. 7A and Supplementary Tables S8, S9). Out of these DEGs, KEGG pathways of photosynthesis-antenna proteins, photosynthesis, nitrogen metabolism, and carbon fixation in photosynthetic organisms were significantly enriched in the green-yellow module (Fig. 7B, Supplementary Table S10), suggesting that the green-yellow module should be associated with yield heterosis. The GO terms of the green-yellow module were significantly enriched in GO:0015979 (photosynthesis; FDR = 3.6E−07), GO:0019684 (photosynthesis, light reaction; FDR = 5.8E−03), and GO:0009765 (photosynthesis, light-harvesting; FDR = 1.4E−04) (Supplementary Fig. S5). The DEG expression levels of CY6203 were inhibited first and then increased in pathways of photosynthesis-antenna proteins and photosynthesis compared with its parents (Supplementary Figs. S6 and S7). In contrast, the DEG expression levels of CY6203 in pathways of nitrogen metabolism and carbon fixation were continuously higher than those of its parents in photosynthetic organisms of all three stages, while their increase gradually decreased, especially in charge of carbonic an hydrase [EC:4.2.1.1] (LOC_Os01g45274, LOC_Os04g33660, and LOC_Os08g36680) and ribulose-bisphosphate carboxylase small chain [EC:4.1.1.39] (LOC_Os12g17600, LOC_Os12g19381, and LOC_Os12g19470) (Supplementary Fig. S8).

Interestingly, the nitrogen metabolism pathway was also identified in the yellow module (FDR = 0.02), including 9 enriched DEGs. Of these DEGs, 5 known genes, *PSR1*^15^, *OsNRT2.3*^16,17^, *OsNRT2.4*^18^, *OsGS1;2*^19–21^ and *OsGS2*^22,23^, carried variants between CH3203 and C106B (Supplementary Table S9). The mRNA editing ratios of these genes were an average of approximately 0.5 in CY6203 (Supplementary Table S5). Their expression levels were higher in CY6203 than C106B and were approximately equivalent to that of CH3203 at the middle filling stage (Supplementary Table S6).

### Comparison of cloned gene expression between Chuanyou6203 and its parents related to yield, quality and KEGG pathway

**Figure 8:**
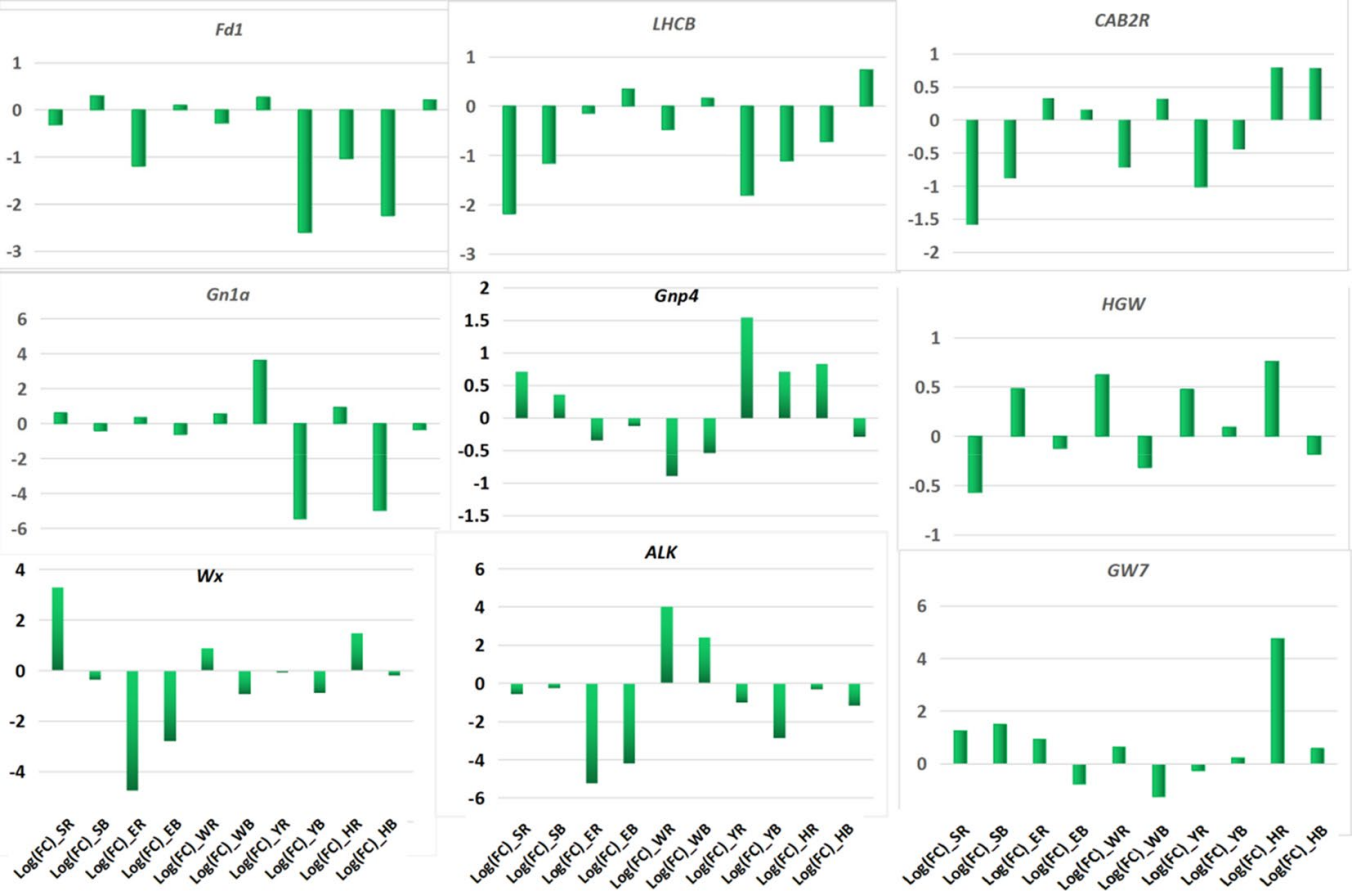
Partial cloned gene expression level of CY6203 compared with its parents using Log_2_ fold change (Log_2_FC_CY6203/CH3203 or C106B_). Log(FC)_SR represents Log_2_FC_CY6203/CH3203_ of panicle samples7 days before heading. Log(FC)_SB represents Log_2_FC_CY6203/C106B_ of panicle samples7 days before heading. Log(FC)_ER represents Log_2_FC_CY6203/CH3203_ of panicle samples 3 days after heading. Log(FC)_EB represents Log_2_FC_CY6203/C106B_ of panicle samples in 3 days after heading. Log(FC)_WR represents Log_2_FC_CY6203/CH3203_ of panicle samples 15 days after heading. Log(FC)_WB represents Log_2_FC_CY6203/C106B_ of panicle samples in 15 days after heading. Log(FC)_YR represents Log_2_FC_CY6203/CH3203_ of sward leaf samples 7 days before heading. Log(FC)_YB represents Log_2_FC_CY6203/C106B_ of sward leaf samples7 days before heading. Log(FC)_HR represents Log_2_FC_CY6203/CH3203_ of sward leaf samples 3 days after heading. Log(FC)_HB represents Log_2_FC_CY6203/C106B_ of sward leaf samples3 days after heading.

According to the annotation of the cloned genes (Fig. 3), we listed the Log_2_FC_CY6203/CH3203 or C106B_ values of cloned genes related to the detected KEGG pathways, yield, and quality in Supplementary Table S11. Of these genes, the expression levels of some genes of CY6203 were higher than that of C106B and lower than that of CH3203 at three stages, such as *HGW*^24^ (Fig. 8), while some genes exhibited the opposite phenotype, such as *Gn1a*^25^. Some were synchronously higher than that of its parents at the booting stage, and then synchronously lower than that of its parents at the middle filling stage, such as *Gnp4*^26^. The expression level of most genes related to chlorophyll was higher in CY6203 than that of C106B, but lower than that of CH3203 at the middle filling stage, for example, *Fd1*, *LHCB*, and *CAB2R*. While the genes related to grain quality were opposite, such as *Wx*^27–29^ and *GL7*^30^. The expression level of *ALK*^31,32^ was higher in CY6203 than in its parents at the middle filling stage (Fig. 8), which might be the main reason why CY6203 showed over-parent heterosis in ASV (Fig. 2).

## Discussion

The use of crop heterosis has notably helped to ensure world food security. For over a hundred years, researchers have been exploring the molecular mechanism of heterosis, starting from the previously proposed hypotheses of dominance, over-dominance, and epistasis to later transcriptome analysis at gene expression levels^1^. It is generally believed that the DEGs between CY6203 and its parents can control the heterosis of the hybrid^2,3,6–8^. Wei et al. identified 3,926 DEGs between LYP9 and its parents and between the parents, 93-11, and PA64s^3^. Zhang et al. indicated that polymorphic promoter *cis*-regulatory elements were closely correlated with DEGs with an additive, as well as over- and under-dominance, gene action^33^. Huang et al. believed that numerous superior alleles contributed to heterosis, but only a small number of loci with strong overdominance affected heterosis in hybrids; for example, *Ehd1*, *Hd1*, *Ghd7*, and *OsSoc1* strongly affected heading date, and *Gn1*, *Ghd7,* and *OsSPL14* affected grain number, and *qSW5* and *Wx* affected chalky grain rate^4^. Chen et al. also believed that the overdominant effect was a major contributor to the grain number heterosis of WFYT025^5^.

Therefore, heterozygosity is generally a prerequisite for gene expression and phenotypic variation in hybrids^1^. In this study, CY6203 with excellent quality and high yield showed visibly overparent heterosis in SPW/D (OMPH:8.85%; OFPH:14.42%), ANSPP (OMPH:27.77%; OFPH:18.64%), AC (OMPH:44.63%; OFPH:6.71%) and ASV (OMPH:6.15%; OFPH:15%). A total of 7473 heterozygous genes in CY6203 were identified due to variant sites between CH3203 and C106B (Supplementary Table S5). In the results of mRNA editing (Supplementary Table S5), transcripts of 14,979 genes from both parents were simultaneously expressed in CY6203, and approximately 40.61% of mRNA editing ratios were between 0.4 and 0.6 (Fig. 5). Interestingly, 1.68% of mRNA editing events (editing ratio ≥ 0.8) in CY6203 favoured one of its parents at three stages or a particular stage, suggesting that the hypothetical heterosis mechanism of CY6203 might involve dominance or epistasis. For example, the average 0.8376 of *GL7*^30^ type belonged to CH3203 type at three stages, and 0.8576 of the *Edh2* type belonged to CH3203 type at only the booting stage. We also noticed that the DEG number of CY3203 vs. CY6203 (7617 DEGs, 4582 upregulated) substantially exceeded that of C106B vs. CY6203 (1164 DEGs, 829 upregulated) in panicles at the booting stage, but at the middle filling stage, the number of DEGs was quite the opposite (4515 and 1658 vs. 1738 and 868). These results indicated that CH3203 and CY106B together contributed to the high yield and good quality of CY6203.

**Figure 9:**
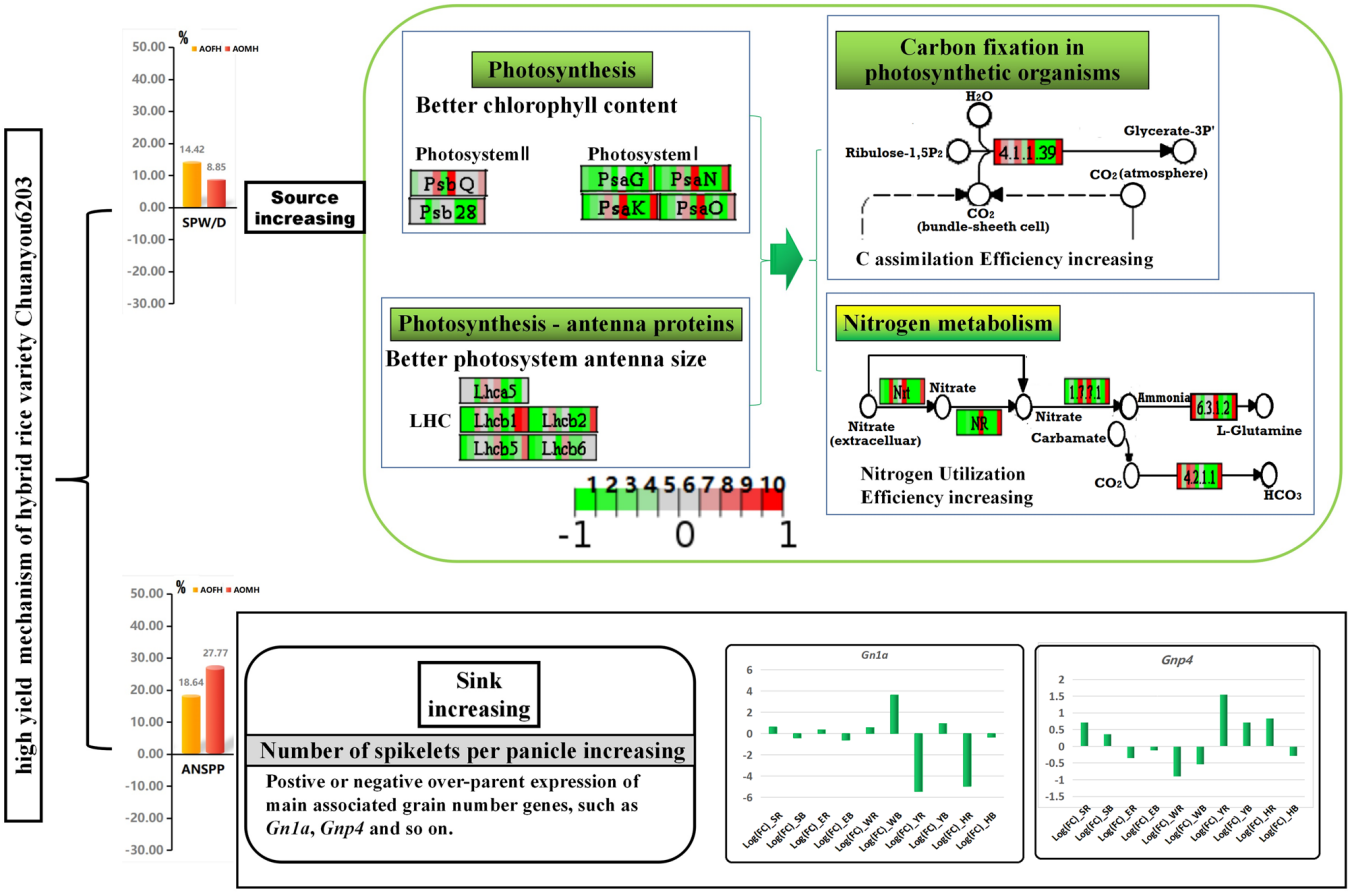
Predicted high yield mechanism of hybrid variety Chuanyou6203. the Green colour represents pathway in the green-yellow module. the yellowish-green gradual discoloration represents the pathway of nitrogen metabolism in the green-yellow and yellow module. The green–red gradual discoloration represents the gene expression level of F_1_, Chuanyou6203 compared with its parents. The green arrow represents negative regulation, and 1 and 2 represent panicle samples 7 days before heading. 3 and 4 represent panicle samples 3 days after heading. 5 and 6 represent panicle samples 15 days after heading. 7 and 8 represent sword leaf samples 7 days before heading. 9 and 10 represent sword leaf samples 3 days after heading. SPW/D: single plant weight per day; ANSPP: average number of spikelets per panicle.

To explain the genetic mechanism of high yield and quality of CY6203, we summarized the following two points. First, the DEGs in the green-yellow module mainly contributed to the increase in the source of CY6203 due to an increase in photosynthetic efficiency and nitrogen utilization efficiency, and a small number of DEGs related to the grain number added spikelet number per panicle amplified its sink (Fig. 9). OMPHs for SPW/D and ANSPP were 8.85% and 27.77%, respectively. Similarly, OFPHs for SPW/D and ANSPP were 14.42% and 18.64%, respectively (Figs. 2, 9). Epigenetic regulation of circadian-mediated changes in chlorophyll biosynthesis and starch metabolism is a direct reason for growth vigour in plant hybrids^1^. Further studies showed that an appropriate reduction in the size of the chlorophyll antenna or chlorophyll content can improve the photosynthetic CO_2_ uptake rates and nitrogen utilization efficiency^34–37^. Of the 298 DEGs in the green-yellow module, 31 DEGs were significantly enriched in pathways of photosynthesis-antenna proteins, photosynthesis, nitrogen metabolism, carbon fixation in photosynthetic organisms, glyoxylate, and dicarboxylate metabolism and linoleic acid metabolism (Supplementary Table S8). In the biological process, photosynthesis negatively regulated light-harvesting (Supplementary Fig. S5). The pathway of nitrogen metabolism was also identified from the yellow module. Of the five cloned genes, *PSR1*^15^ encoded a nitrite reductase with nitric acid assimilation, *OsNRT2.3*^16,17^ and *OsNRT2.4*^18^ were inferred to control regulation of rice high-affinity nitrate transport, and *OsGS1;2*^19–21^ and *OsGS2*^22,23^ showed coexpression affecting nitrogen metabolism and plant growth. In summary, we speculate that the photosynthetic CO_2_ uptake rates and nitrogen utilization efficiency are improved in CY6203 due to its sustained better chlorophyll accumulation and suitable size of the photosynthesis antenna from the booting to the middle filling stage (Fig. 9). Therefore, these genes were coexpressed, which increased nitrogen utilization efficiency and carbon dioxide absorption from the booting to the middle filling stage in CY6203. Thus, the coordinated expression of DEGs in the green-yellow module can improve the photosynthetic efficiency and nitrogen utilization efficiency of CY6203.

Among 1079 cloned genes with different alleles between CH3203 and C106B, 15 genes were related to yield trait-based previous reports at https://www.ricedata.cn/ (Fig. 3). Only *SPP1* was inferred to control the number of spikelets per panicle^38^. The expression level of CY6203 was higher than that of its parents at the booting stage (Supplementary Table S11). However, the gene (new name: *OsJAR2*) was determined to be involved in the wound- and pathogen-induced jasmonic acid signalling^39^. Although two well-known genes controlling the grain number per panicle, *Gn1a*^25^ and *Gnp4*^26,40^, without variants between CH3203 and C106B, showed middle-parent and over-parent expression levels in CY6203 at the booting stage, respectively (Supplementary Table S11). Ashikari et al.^25^ reported that the decrease in the activity of *Gn1a* increased the spikelet number. Similarly, increasing the expression level of *Gnp4* also increased the spikelet number^26^. Therefore, we infer that the coregulation of *Gn1a* and *Gnp4* jointly increases the average number of spikelets per panicle, resulting in the amplified sink of CY6203.

**Figure 10:**
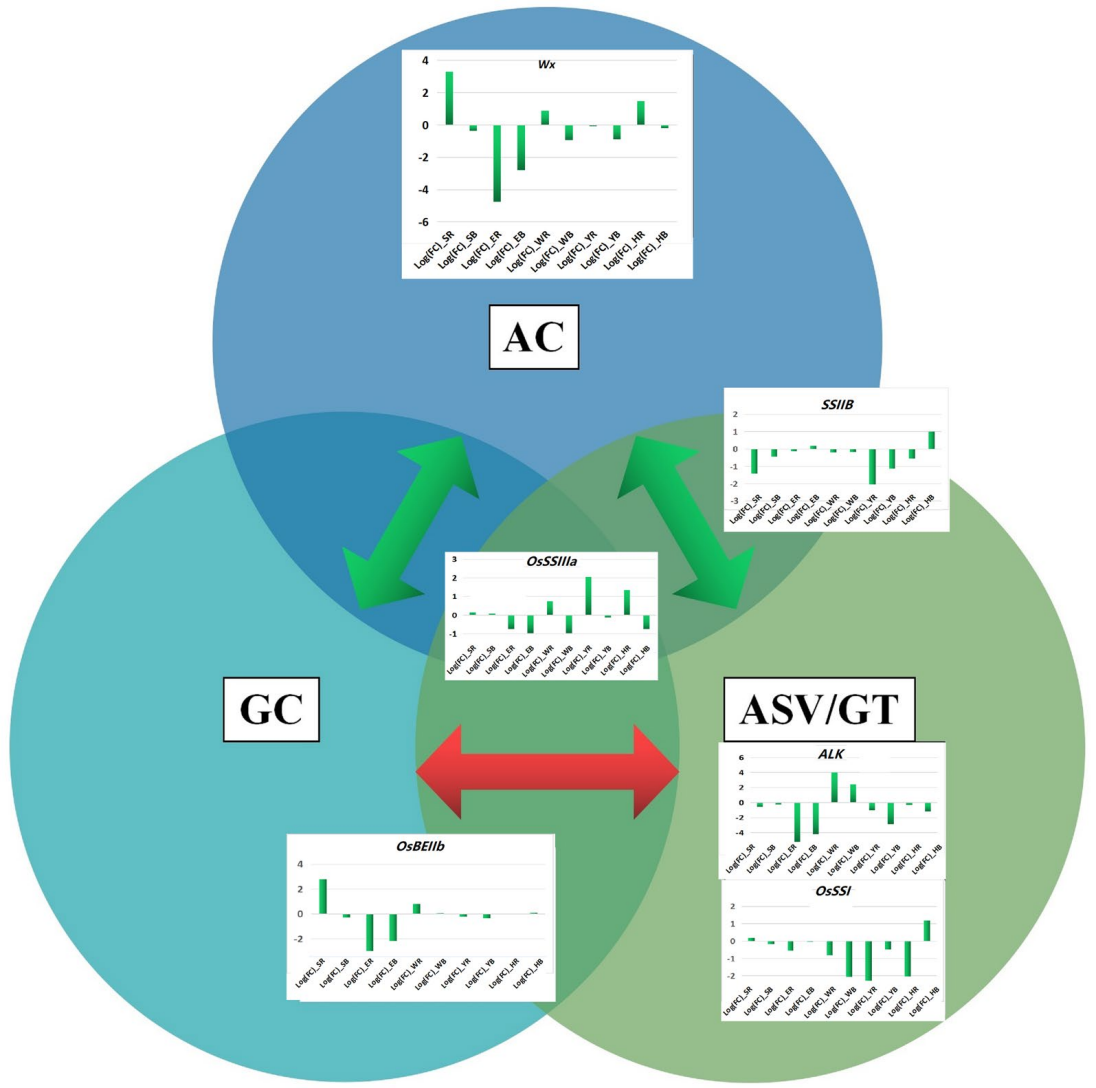
Predicted quality mechanism of hybrid variety Chuanyou6203 based on previous reports. The green arrows represent negative regulation. The red arrow represents positive regulation.

Second, the balanced expression of the major high-quality alleles of C106B and CH3203 in CY6203 contributed to the outstanding quality of CY6203. At the middle filling stage, the number of DEGs between CY6203 and C106B was greater than that of DEGs between CY6203 and CH3203. However among coexpressed DEGs, the ratio of upregulated DEGs between CY6203 and CH3203 was greater than that of DEGs between CY6203 and C106B (Fig. 5A), which might indicate the balanced expression of some related quality genes of C106B and CH3203 in CY6203. Complex genetic networks that affect AC, GC, and gelatinization temperature (GT) determine the cooking and eating quality (ECQs) of rice^41^ (Fig. 10). Among these genes, *Wx* and *SSII-3* are the two essential genes that affect AC, GC, and GT. Different alleles of *Wx* lead to regional variation in rice AC and have affected consumer preferences^27–29,41–43^. The substitution of bases in the coding region of the *ALK* gene may change the crystal structure of amylopectin, which may lead to a change in ASV in rice^31^. By changing the expression level of *OsBEIIb*^44,45^ in rice endosperm, GC can be manipulated due to the change in starch structure. AC is significantly negatively correlated with GC and ASV/GT, and GC is significantly positively correlated with GT^41^. However, in the quality traits of CY6203, the increase in the ASV value did not lead to an increase in the GC value, and the increase in the AC value did not lead to a decrease in the ASV value (Fig. 2 and Table 1).

Genome comparison results showed that there were SNP/InDel variants in the four major quality-related genes between CH3203 and C106B, including *SSIIB*, *Wx*^46,47^, *OsSSI*^48^, and *OsSSIIIa*^49^. The *Wx* alleles of CH3203 and C106B both belonged to the *Wx*^*b*^ type of *japonica* rice, except for a nucleotide deletion in the intronic sequence of the *Wx* gene between CH3203 and C106B (Supplementary Table S2). Compared with CH3203, there were three single-nucleotide deletions, one single-nucleotide insertion, and one SNP in the *OsSSI* sequence of C106B. There were six InDels and 12 SNPs in the *OsSSIIIa* sequence between CH3203 and C106B. We noticed that the average mRNA editing ratios of *Wx*, *OsSSI*, and *OsSSIIIa* were 0.4412, 0.4679 and 0.5255 in CY6203 at the booting, flowering, and middle filling stages, respectively, which indicated that these genes expressed additive effects. However, the expression levels of *Wx* and *OsSSIIIa* in CY6203 were upregulated compared with CH3203, and was downregulated compared with C106B at the middle filling stage, which may lead to increased AC content in CY6203. The expression level of *OsSSI* in CY6203 was downregulated, and *ALK* was upregulated compared with its parents at the middle filling stage (Fig. 10), which may be the reason for the increase in the ASV value in CY6203. In addition, the decrease in *OsSSI* gene expression resulting in the decrease insoluble starch content may be an important reason for the decrease in GC value in CY6023. Therefore, we believed that superior starch synthesis alleles from C106B and CH3203 balanced expression contributed to the outstanding quality of CY6203.

## Conclusions

In summary, we systematically analysed the transcriptome and genome profiles from hybrid rice, Chuanyou6203 (CY6203), and its parents by RNA-sequencing and resequencing. A total of 7,473 different genes were found between CH3203 and C106B. A total of 436, 70, 551, 993, and 1,216 common DEGs between CY6203 and both of its parents were identified at the same stage in panicles and flag leaves. Of the common DEGs, the numbers of upregulated DEGs between CY6203 and CH3203 were all greater than those of upregulated DEGs between CY6203 and C106B in panicles and flag leaves at the booting, flowering, and middle filling stages. In addition, approximately 40.61% of mRNA editing ratios were between 0.4 and 0.6, and 1.68% of mRNA editing events (editing ratio ≥ 0.8) in CY6203 favoured one of its parents at three stages or a particular stage, suggesting that the hypothetical heterosis mechanism of CY6203 might involve dominance or epistasis. The results of WGCNA, KEGG pathway and GO enrichment analysis showed that the green-yellow module was inferred to be associated with yield heterosis including KEGG pathways of photosynthesis-antenna proteins, photosynthesis, nitrogen metabolism, and carbon fixation in photosynthetic organisms. The balanced expression of the major high-quality alleles of C106B and CH3203 in CY6203 contributed to the high quality of CY6203. Our study offers a new data set that may help to elucidate the molecular mechanism governing yield heterosis and the formation of a high quality hybrid rice variety.

## Materials and methods

### Plant materials

Chuanyou6203 (CY6203) is an outstanding hybrid rice variety with excellent quality and high yield, stained from a cross between the Wild Abortive Cytoplasmic Male Sterility (WA-CMS) line Chuan106A (C106A) and the restorer line Chenghui3203 (CH3203). Chuan106B is the isonuclear fertile line of Chuan106A. CY6203 was released in 2011 and 2014 by the Variety Certification Committee of Sichuan province and the Country Variety Certification Committee in the rice zone of the upper Yangtze River, respectively.

### Phenotyping and sampling

CY6203, CH3203, and Chuan106B (C106B) (the isonuclear fertile line of Chuan106A) were grown in a rice paddy at the experimental field of the Sichuan Academy of Agricultural Sciences. To ensure sampling at the same time, CY6203, CH3203, and C106B were sown on April 5, 10, and 25, 2019, respectively. The 30-day seedlings were transplanted with a spacing of 17 cm × 25 cm, with three replications. Each material was planted in five rows with 10 plants per row. Field management followed normal procedures for rice. These three lines were selected in this study for the measurement of five agronomic traits. The five agronomic traits were single plant weight per day (SPW/D, in grams/plant/day) = single plant weight/days of maturity, plant height (PH, in centimetres), panicle length (PL, in centimetres), the average number of spikelets per panicle (ANSPP), and 1000-gain weight (TGW, in grams). For each material, 500 g grains were sent to the Rice Product Quality Inspection & Supervision Testing Center of Ministry of Agriculture and Rural Affairs to measure quality traits with three replications. They were head rice ratio (HRR, the ratio of head milled rice), grain length/grain width (L/W), amylose content (AC), alkali spreading value (ASV), and gel consistency (GC). The means of replications for each trait were used to calculate the over-male-parent heterosis (OMPH) and over-female-parent heterosis (OFPH) of the F_1_ hybrid CY6203. The formulas were as follows:

$$ \begin{aligned} {\text{Over-male-parent}}\,{\text{heterosis}}\,\left( {{\text{OMPH}}} \right)\left( \% \right) & = ({\overline{\text{F}}}_{1} - \overline{{{\text{MP}}}} )/\overline{{{\text{MP}}}} \times {1}00\% \\ {\text{Over-female-parent}}\,{\text{heterosis}}\,\left( {{\text{OFPH}}} \right)\left( \% \right) & = ({\overline{\text{F}}}_{1} - \overline{{{\text{FP}}}} )/\overline{{{\text{FP}}}} \times {1}00\% \\ \end{aligned} $$

The $\overline{{{\text{F}}_{{1}} }}$ was the mean of replications, for each trait for CY6203. The $\overline{{{\text{HP}}}}$ was the mean of replications for each trait, for CH3203. The $\overline{{{\text{FP}}}}$ was the mean of replications, for each trait for C106B.

The first samples were collected seven days before heading, including flag leaves and panicles. The flag leaf samples were named Y6203, Y3203, and Y106B, and the panicle samples were named S6203, S3203, and S106B, respectively. The second samples, including flag leaves and panicles, were taken 3 days after heading. The flag leaf samples were named H6203, H3203, and H106B, and the panicle samples were named E6203, E3203, and E106B, respectively. The third panicle sampling was conducted 15 days after heading. The panicle samples were named W6203, W3203, and W106B. Each sampling time was 9:00–10:00 with three replications. All samples were stored at -80℃ for genomic resequencing and RNA-Seq analysis.

### DNA, total RNA isolation and sequencing

The total DNA of the two parents was extracted with the Plant Genome DNA Extraction kit. The total RNA of each sample tissue was extracted with the RNA prep Pure Plant Kit with RNase-Free DNase. The quality and integrity of DNA and RNA were tested using the Agilent Bioanalyzer 2100 system (Agilent, Santa Clara, CA, USA). All DNA and RNA samples were sent to Gene Denovo Biotechnology Co., Ltd. (Guangzhou, China) for sequencing. The DNA/cDNA libraries were sequenced using Illumina HiSeq 4000 by Gene Denovo Biotechnology Co., Ltd.

### Identification of variations between CH3203 and C106B

Based on the Nipponbare reference genome MSU 7, the SNP/InDel variants between CH3203 and C106B were identified and filtered (MAPQ ≥ 25) by SAMtools^50^. ANNOVAR^51^ was used for the SNP/InDel annotation. Then, by comparing the variant types of CH3203 and C106B at the same position, different variant type sites were selected, and the number of different SNP/InDel per 200 kb was calculated. RIdeogram^52^ was used to visualize and localize the density distribution of different SNP/InDel densities and cloned genes related to yield, quality, and some KEGG pathways in the whole genome.

### Read mapping and analysis

The Nipponbare reference genome MSU7 and its annotation file were downloaded from the Rice Genome Annotation Project (https://rice.plantbiology.msu.edu/)^53^. First, we used FastQC software to evaluate the quality of the raw reads. To obtain high-quality clean reads, the raw reads were further filtered according to the following rules: 1) Delete the reads containing the adapters. 2) Remove the reads containing more than 10% of unknown nucleotides (N). 3) Remove low-quality reads containing more than 50% of low-quality (Q-value ≤ 20) bases. Second, the RNA-seq clean reads were aligned to the ribosomal RNA (rRNA) database using Bowtie2^54^, and then the rRNA-mapped reads were removed. Third, the remaining reads of CH3203 and C106B were mapped to the Nipponbare reference genome by Bowtie2^54^. The alignment parameters were as follows: (1) maximum read mismatch was 2; (2) the error of distance between mate-pair reads was ± 80 bp; and (3) the distance between mate-pair reads was 50 bp. Fourth, the transcripts were detected by the Reference Annotation Based Transcript method using Cufflinks software^55^. Fifth, gene abundances were quantified by RSEM software^56^. The gene expression level was normalized using the fragments per kilobase of transcript per million mapped reads (FPKM) method. The edgeR package^57^ was applied to identify differentially expressed genes (DEGs) among all samples. Outlier samples were examined using hierarchical clustering and removed.

To characterize common DEGs between CY6203 and its parents, Venn diagrams were drawn by https://bioinformatics.psb.ugent.be/webtools/Venn/.

Finally, RNA editing was also analysed. RNA editing referred to variants on the mRNA level through InDel or base substitutions^58,59^. The reliable RNA editing sites from SNP sites were screened according to the following steps^60,61^: (1) SNPcalling was carried out by SAMtools (https://www.htslib.org/doc/samtools.html)^50^; (2) correction of the SNPs around the InDel region; (3) selection of non-overlapping SNPs in UTR and exon region; (4) selection of SNPs with reference reads ≥ 2 and variant reads ≥ 3; (5) selection of SNPs with the mRNA editing ratios between 0.1000 and 0.9000; and (6) counting the frequency of SNPs per interval ratio of 0.1000 in each material, and calculating the mean of mRNA editing loci.

Raw sequence data reported in this paper have been deposited (PRJCA003209) in the Genome Sequence Archive^62^ in the BIG Data Center^63^, Chinese Academy of Sciences under accession code ‘CRA003262’ for C106B and CH3203 genome sequencing data and CY6203 transciptome sequencing data that are publicly accessible at https://bigd.big.ac.cn/gsa.

### Quantitative real-time PCR (qRT-PCR) analysis

Eighteen DEGs detected by RNA-seq were selected for validation by qRT-PCR. Total RNA was extracted from forty-five samples using an RNA Prep Pure Plant Kit containing RNase-Free DNase. Two micrograms of total RNA was reverse transcribed using the HiScript First Strand cDNA Synthesis Kit (Vazymebiotech Co., Ltd., Nanjing, China). SYBR-based qRT-PCR analyses were performed on ABI Step One Plus according to the protocol (ChamQ SYBR qPCR Master Mix) provided by the manufacturer (Vazymebiotech Co., Ltd., Nanjing, China). All qRT-PCR reactions were biologically repeated 3 times, and the results were analysed based on the 2^−△△Ct^ method using the system’s relative quantification software (version 1.5). The cycle threshold values for each gene were normalized against the expression level of the rice *Actin* gene with the primer sequences 5′-CCACTATGTTCCCTGGCATT-3′ and 5′-GTACTCAGCCTTGGCAATCC-3′. All primers used for qRT-PCR in this study are listed in Supplementary Table S12.

### Weighted gene coexpression network analysis

To identify DEGs between CY6203 and its parents, we identified genes with a Log_2_fold change > 1 or < − 1, and a false discovery rate (FDR) < 0.05 in comparison as significant DEGs^64^. The weighted gene coexpression network analysis (WGCNA) package^14^ was used to infer the DEG modules of the co-expression network. DEGs were filtered before coexpression network inference to eliminate genes not expressed in more than 10% of samples or that showed variance = 0 across samples. The automatic one-step network construction and module detection method with default settings were carried out, including an unsigned type of topological overlap matrix (TOM), a power β of 18 (R Squared Cut = 0.85), a minimum module size of 30, and a branch merge cut height of 0.25. The module eigengene value was calculated and used to test the association of each module with each of 43 samples. Heatmaps for the gene expression and characteristic bar graphs of each module were drawn. Moreover, the eigengene adjacency cluster and corresponding heatmap were visualized based on eigengene-based intramodular connectivity measures (kME).

### KEGG pathway and GO enrichment analysis of WGCNA modules

To further clarify the biological functions of each module between F_1_ (CY6203) and its parents (C106B and CH3203), the genes of each WGCNA module were previously subjected to KEGG pathway enrichment analysis by ExPath 2.0 (https://expath.itps.ncku.edu.tw/)^65^ and GO enrichment analysis by agriGO v2 (https://systemsbiology.cau.edu.cn/agriGOv2/)^66^. The significance level was FDR ≤ 0.05. The enrichment results were visualized by ggPlot2^67^. The expressed pattern of DEGs involved in each enriched pathway of the green-yellow module was visualized using the Log2(fold change) value by Pathview Web (https://pathview.uncc.edu/)^68,69^.

### Fold change comparison of yield, quality, and KEGG related genes

Yield-, quality-, and KEGG pathway-related genes were selected based on previous reports at https://www.ricedata.cn/ and their annotated information in the funRiceGenes database (https://funricegenes.github.io/). We calculated Log_2_FC_CY6203/CH3203_ and Log_2_FC_CY6203/C106B_ of these genes for each sample tissue of CY6203. We made a definition for a calculating formula:

$$ {\text{Log}}_{{2}} \,{\text{fold}}\,{\text{change}}\,({\text{Log}}_{{2}} {\text{FC}}_{{{\text{CY62}}0{3}/{\text{CH32}}0{\text{3 or C1}}0{\text{6B}}}} ) = {\text{Log}}_{{2}} \left( {\frac{{\overline{{{\text{FPKM}}}}_{{{\text{CY}}6203}} }}{{\overline{{{\text{FPKM}}}}_{{{\text{CH}}3203{\text{ or C}}106{\text{B}}}} }}} \right) $$

where the $\overline{{{\text{FPKM}}}}_{{{\text{CY}}6203}}$ meant is the mean FPKM value of a gene in the CY6203 transcript. The $\overline{{{\text{FPKM}}}}_{{{\text{CH}}3203\,{\text{or}}\,{\text{C}}106{\text{B}}}}$ is the mean FPKM value of a gene in the CH3203 or C106B transcript.

## Supplementary information

Supplementary Figure S1.

Supplementary Figure S2.

Supplementary Figure S3.

Supplementary Figure S4.

Supplementary Figure S5.

Supplementary Figure S6.

Supplementary Figure S7.

Supplementary Figure S8.

Supplementary Figure S9.

Supplementary Table S1.

Supplementary Table S2.

Supplementary Table S3.

Supplementary Table S4.

Supplementary Table S5.

Supplementary Table S6.

Supplementary Table S7.

Supplementary Table S8.

Supplementary Table S9.

Supplementary Table S10.

Supplementary Table S11.

Supplementary Table S12.

## Abbreviations

AC: Amylose content
ANSPP: Average number of spikelets per panicle
ASV: Alkali spreading value
C106B: Chuan 106B
CH3203: Chuanhui3203
CY6203: Chuanyou6203
DEGs: Different expression genes
ECQ: The cooking and eating quality
FC: Fold change
FDR: False discovery rate
FPKMs: Fragments per kb for a million reads
GC: Gel consistency
GO: Gene ontology
HRR: Head rice ratio
KEGG: Kyoto encyclopedia of genes and genomes
kME: Eigengene-based intramodular connectivity measures
L/W: Grain length/grain width
MSU 7: MSU RICEGENOMEANNOTATIONPROJECT RELEASE 7
OFPH: Over-female-parent heterosis
OMPH: Over-male-parent heterosis
PH: Plant height
PL: Panicle length
qRT-PCR: Quantitative real-time polymerase chain reaction
TGW: 1000-gains weight
SPW/D: Single plant weight per day
WA-CMS: Wild abortive cytoplasmic male sterility
WGCNA: The weighted gene coexpression network analysis
