# Supplementary material for: Transcriptome and genome sequencing elucidates the molecular basis for the high yield and good quality of the hybrid rice variety Chuanyou6203
Source: Sci Rep. 2020 Nov 17;10:19935. doi: 10.1038/s41598-020-76762-3 (PMC7673993; doi:10.1038/s41598-020-76762-3)
Supplement: Supplementary file 3 — Supplementary Figure S3. [file 41598_2020_76762_MOESM3_ESM.pdf]

Transcriptome and genome sequencing elucidates the molecular basis for the high yield and good quality of the hybrid rice variety Chuanyou6203

Juansheng Ren[1]*， Fan Zhang[2]*， Fangyuan Gao[1], Lihua Zeng[3]，Xianjun Lu[1], Xiuqin Zhao[2], Jianqun Lv[1], Xiangwen Su[1], Liping Liu[1], Mingli Dai[1], Jianlong Xu[2], Guangjun Ren[1]**

[1]Crop Research Institute, Sichuan Academy of Agricultural Sciences, Chengdu, 610066, P.R. China

[2]Institute of Crop Sciences, Chinese Academy of Agricultural Sciences, Beijing, 100081, P.R. China

[3]Sichuan Normal University, Chengdu, 610066, P.R. China

*These authors contributed equally to the work

**Corresponding author email: guangjun61@sina.com.

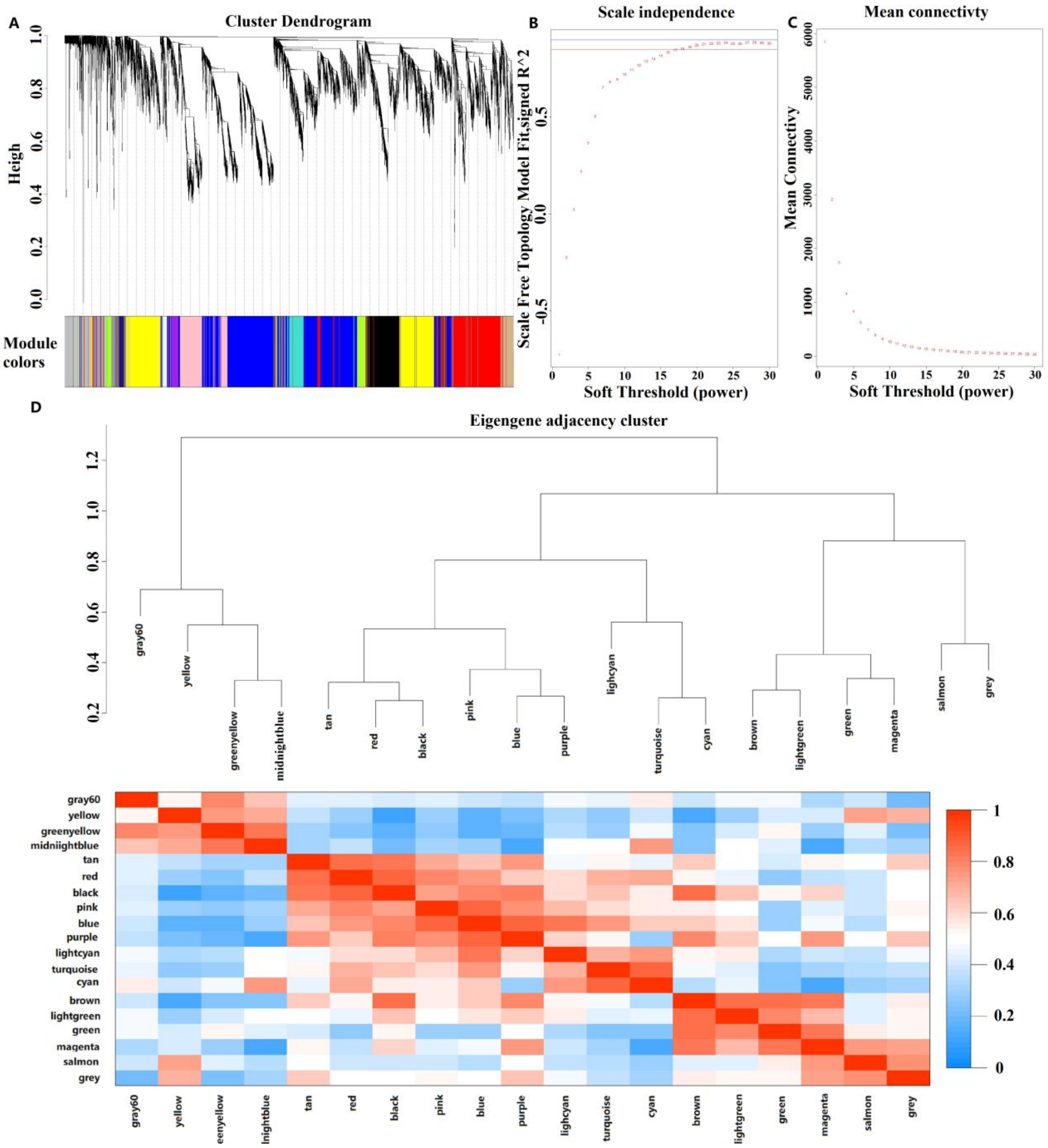

Figure S3 Weighted gene coexpression network analysis (WGCNA) of the DEGs among Chuanyou6203, Chenghui3203 and Chuan106B. A: Hierarchical cluster analysis of all DEGs models based on expression data. B and C: Selecting β, balancing the topology criterion and mean connectivity. D: Cluster and heatmap of meta-modules adjacency by eigengene.
