# Supplementary material for: Transcriptome and genome sequencing elucidates the molecular basis for the high yield and good quality of the hybrid rice variety Chuanyou6203
Source: Sci Rep. 2020 Nov 17;10:19935. doi: 10.1038/s41598-020-76762-3 (PMC7673993; doi:10.1038/s41598-020-76762-3)
Supplement: Supplementary file 8 — Supplementary Figure S8. [file 41598_2020_76762_MOESM8_ESM.pdf]

Transcriptome and genome sequencing elucidates the molecular basis for the high yield and good quality of the hybrid rice variety Chuanyou6203

Juansheng Ren[1]*， Fan Zhang[2]*， Fangyuan Gao[1], Lihua Zeng[3]，Xianjun Lu[1], Xiuqin Zhao[2], Jianqun Lv[1], Xiangwen Su[1], Liping Liu[1], Mingli Dai[1], Jianlong Xu[2], Guangjun Ren[1]**

[1]Crop Research Institute, Sichuan Academy of Agricultural Sciences, Chengdu, 610066, P.R. China

[2]Institute of Crop Sciences, Chinese Academy of Agricultural Sciences, Beijing, 100081, P.R. China

[3]Sichuan Normal University, Chengdu, 610066, P.R. China

*These authors contributed equally to the work

**Corresponding author email: guangjun61@sina.com.

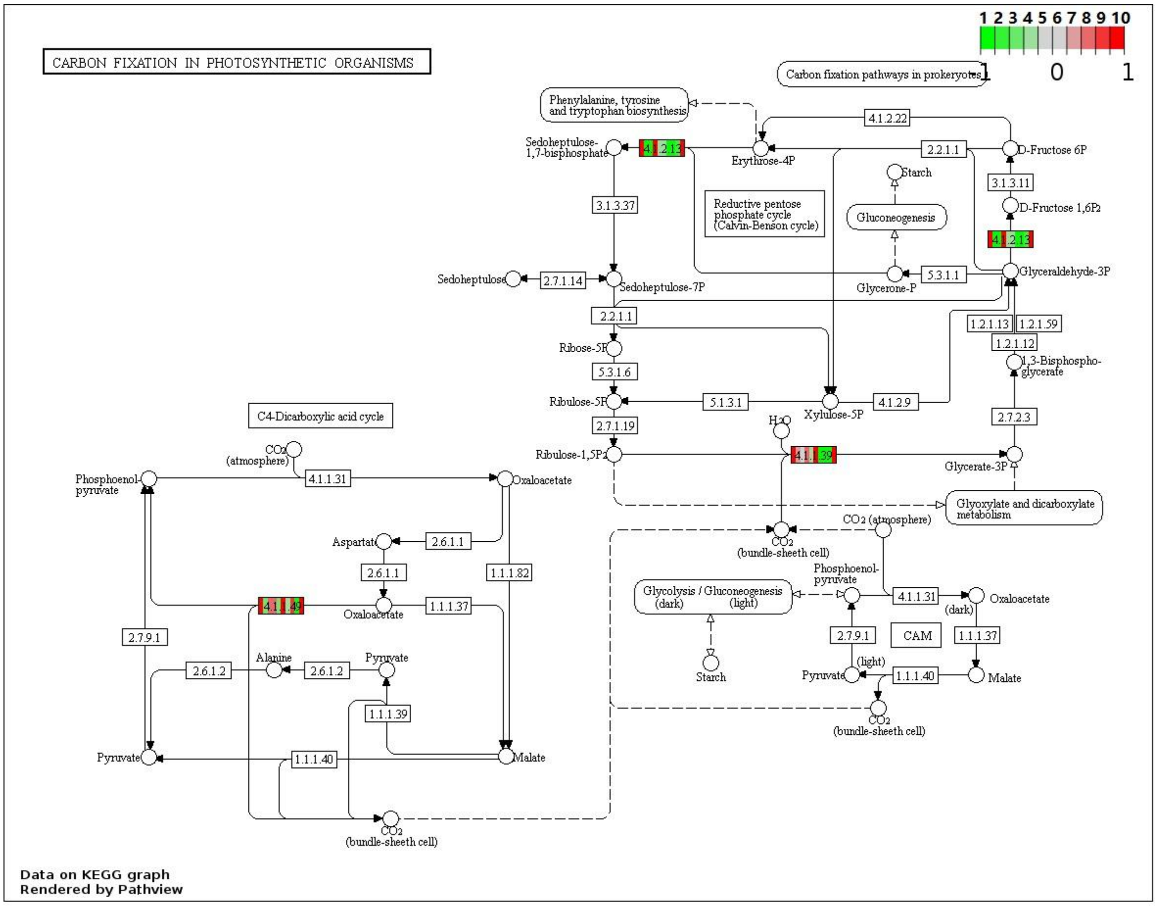

Figure S8. DEGs belonging to green-yellow modules enrich the pathway of carbon fixation in photosynthetic organisms by PATHVIEW Wed (https://pathview.uncc.edu/). 1 and 2 represent panicle samples 7 days before heading. 3 and 4 represent panicle samples 3 days after heading. 5 and 6 represent panicle samples 15 days after heading. 7 and 8 represent sword leaf samples 7 days before heading. 9 and 10 represent sword leaf samples 3 days after heading.
