# Supplementary material for: Transcriptome and genome sequencing elucidates the molecular basis for the high yield and good quality of the hybrid rice variety Chuanyou6203
Source: Sci Rep. 2020 Nov 17;10:19935. doi: 10.1038/s41598-020-76762-3 (PMC7673993; doi:10.1038/s41598-020-76762-3)
Supplement: Supplementary file 12 — Supplementary Table S3. [file 41598_2020_76762_MOESM12_ESM.pdf]

Transcriptome and genome sequencing elucidates the molecular basis for the high yield and good quality of the hybrid rice variety Chuanyou6203

Juansheng Ren*[1], Fan Zhang*[2], Fangyuan Gao[1], Lihua Zeng[3]， Xianjun Lu[1], Xiuqin Zhao[2], Jianqun Lv[1], Xiangwen Su[1], Liping Liu[1], Mingli Dai[1], Jianlong Xu[2], Guangjun Ren[1]**

[1]Crop Research Institute, Sichuan Academy of Agricultural Sciences, Chengdu, 610066, P.R. China

[2]Institute of Crop Sciences, Chinese Academy of Agricultural Sciences, Beijing, 100081, P.R. China

[3]Sichuan Normal University, Chengdu, 610066, P.R. China

*These authors contributed equally to the work

**Corresponding author email: guangjun61@sina.com.

Table 3 Summary of RNA-seq data and mapping statistics on the reference genome

Sample	HQ Clean Data (bp)	HQ Clean Reads	Q30 %	GC %	Reads Num. after filtered rRNA	Mapping Ratio %	Known Gene Num.
W6203-1	11300506106	76169578	94.24	54.61	72665108	84.18	25242
W6203-2	8148068869	55160382	89.49	54.22	53297408	78.40	24332
W6203-3	8696052450	59056872	88.80	54.23	56556802	77.95	24381
W3203-1	10053674119	67735144	94.02	55.13	66424844	85.62	24914
W3203-2	12056776812	81251036	93.79	55.37	77712124	85.50	24998
W3203-3	10802879693	72757552	94.13	55.34	69307686	86.56	24860
W106B-1	8981285607	60551516	93.42	54.86	58516764	85.80	24432
W106B-2	9178862093	61853502	93.56	54.30	59283840	85.60	24998
W106B-3	10191169486	68587864	94.28	55.28	66087670	86.30	24856
Y6203-1	11823055437	79781008	93.63	54.08	58017758	83.11	24306
Y6203-2	7671360039	51701644	93.44	54.50	49284946	84.42	25367
Y6203-3	10012576488	67501494	93.68	54.64	63490854	84.69	26083
Y3203-1	12092508354	81491078	93.97	55.91	75947322	85.77	24714
Y3203-2	8408234170	56695606	93.76	55.75	53715170	86.32	24632
Y3203-3	12809600876	86341206	93.75	55.52	82817144	86.61	25311

Y106B-1	11842407174	79770932	93.91	54.85	72457558	85.93	25471
Y106B-2	11178089278	75285346	94.13	54.54	72157014	86.16	25152
Y106B-3	9239609465	62259138	93.91	55.32	59810178	85.70	25016
S6203-1	10730388792	72360936	94.01	55.04	70317550	86.46	29388
S6203-2	9854289411	66464632	93.33	54.62	64489684	85.79	28987
S6203-3	11157621901	75302106	93.28	54.50	73003714	85.92	29462
S3203-1	8972285496	60796168	91.28	54.12	58865464	82.89	28069
S3203-2	8262342135	56020796	90.58	54.71	54750640	81.95	28069
S3203-3	9285781818	63078348	90.14	54.09	61742454	81.16	28145
S106B-1	8869336543	60144702	90.73	54.48	58922058	81.73	28940
S106B-2	10886785432	73975418	90.12	55.22	71669314	80.26	28429
S106B-3	9447720271	63963906	91.14	54.93	61909592	82.37	28588
H6203-1	8029426229	54619306	89.72	54.60	51226864	78.17	25300
H6203-2	7569385291	51355174	90.09	54.34	49506816	79.12	24753
H6203-3	13645344281	92035562	93.84	54.59	87451782	84.14	26178
H3203-1	6505425062	43888162	91.83	56.65	42102810	84.02	23611
H3203-2	8895684668	60425686	89.88	54.94	58176950	79.98	24391
H3203-3	9518667597	64537372	90.45	54.77	62472786	80.86	24647
H106B-1	9811338647	66372186	89.97	54.83	64280094	80.75	25072
H106B-2	8279694311	56215400	90.13	54.51	53725478	79.14	24764
H106B-3	9576682256	64979914	90.18	54.66	61375856	79.19	25265
E6203-1	8683332115	58887944	90.56	54.10	56921326	79.35	27976
E6203-2	8642034305	58747182	88.25	52.68	56772730	77.59	27843
E6203-3	9093034449	61516102	91.25	54.41	59206698	81.40	28003
E3203-1	9426382278	63953574	90.43	54.59	62360898	80.43	28036
E3203-2	10154707165	68779518	91.08	55.23	66544724	81.54	27933
E3203-3	14817194758	101156682	89.88	55.28	97558562	79.23	28608
E106B-1	11487055944	78309042	89.66	54.22	75690020	78.39	28569
E106B-2	12661640296	85381398	94.03	54.72	82166340	84.79	28797
E106B-3	11395592924	76849592	93.88	55.51	74749264	84.79	28622
Mean	10003242019.80	67645949.02	91.99	54.77	64566903.51	82.80	26344.67
Max	14817194758	101156682	94.28	56.65	97558562.00	86.61	29462.00
Min	6505425062.0	43888162.0	88.25	52.68	42102810.00	77.59	23611.00
